# Evaluation of dosimetric advantages of using patient‐specific aperture system with intensity‐modulated proton therapy for the shallow depth tumor

**DOI:** 10.1002/acm2.12231

**Published:** 2017-11-27

**Authors:** Keisuke Yasui, Toshiyuki Toshito, Chihiro Omachi, Kensuke Hayashi, Kenichiro Tanaka, Kumiko Asai, Akira Shimomura, Rie Muramatsu, Naoki Hayashi

**Affiliations:** ^1^ Nagoya Proton Therapy Center Nagoya City West Medical Center Nagoya Japan; ^2^ School of Health Sciences Faculty of Radiological Technology Fujita Health University Toyoake Japan

**Keywords:** aperture, IMPT, lateral penumbra, proton beam, spot scanning

## Abstract

In this study, we evaluate dosimetric advantages of using patient‐specific aperture system with intensity‐modulated proton therapy (IMPT) for head and neck tumors at the shallow depth. We used four types of patient‐specific aperture system (PSAS) to irradiate shallow regions less than 4 g/cm^2^ with a sharp lateral penumbra. Ten head and neck IMPT plans with or without aperture were optimized separately with the same 95% prescription dose and same dose constraint for organs at risk (OARs). The plans were compared using dose volume histograms (DVHs), dose distributions, and some dose indexes such as volume receiving 50% of the prescribed dose (V_50_), mean or maximum dose (D_mean_ and D_max_) to the OARs. All examples verified in this study had decreased V_50_ and OAR doses. Average, maximum, and minimum relative reductions of V_50_ were 15.4%, 38.9%, and 1.0%, respectively. D_max_ and D_mean_ of OARs were decreased by 0.3% to 25.7% and by 1.0% to 46.3%, respectively. The plans with the aperture over more than half of the field showed decreased V_50_ or OAR dose by more than 10%. The dosimetric advantage of patient‐specific apertures with IMPT was clarified in many cases. The PSAS has some dosimetric advantages for clinical use, and in some cases, it enables to fulfill dose constraints.

## 1 INTRODUCTION

In recent years, accelerators, irradiation control systems, and treatment planning systems have been improved and, as a result of these improvements, scanning proton therapy systems have become more widely used.^1^ In spot scanning irradiation, a monoenergetic beam is delivered to a given spot at the target volume specified in the treatment plan and a full scan across the target volume and the dose distribution in the depth direction is formed by multiple monoenergetic beams.^2^ The energy generated by a commercially available accelerator for proton therapy has a lower limit of approximately 4 g/cm^2^ water equivalent penetration because of the stability of the accelerator, beam efficiency, and effective spot size. This limitation complicates the treatment planning so correspond to this limitation. One method to irradiate shallower regions less than 4 g/cm^2^ is to use an energy absorber (EA); however, the monoenergetic beam with an EA has a large spot size due to scattering and has a limited ability to make sharp lateral penumbra, worsening the dose distribution. Although in some cases the scanning method has some issues with the lateral penumbra, it has some advantages over the passive method and x‐ray treatment, and a number of studies using scanning proton beams reported dose comparisons of passive beam and x‐ray beam^3^, ^4^ or verification of the relative biological effectiveness (RBE).^5^ Flexibility of scanning technique allows for various irradiation methods, and these methods can improve the dose distribution. Developments in treatment planning systems (TPS), treatment machines, and treatment techniques permit the use of intensity‐modulated proton therapy (IMPT) for various tumor sites.^6^, ^7^

**Figure 1:**
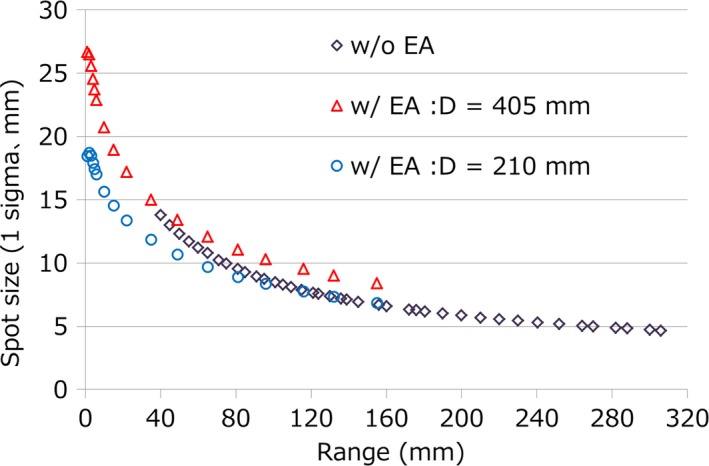
Air spot sizes (1σ) in the plane of the isocenter versus range. Data are measured with (w/) or without (w/o) the EA. D is the distance from the EA to the isocenter.

Figure 1 shows the archived spot sizes with or without EA that were retrieved from the Hitachi ProBeat III operating in the Nagoya Proton Therapy Center (NPTC),^8^ and these data were presented in a previous paper.^9^ Without the EA, the maximum spot size in air was 13.8 mm at the isocenter plane and the minimum range was 4 g/cm^2^. Regions below 4 g/cm^2^ could be irradiated using EA, but the spot size of minimum range was more than 26.7 mm. These bigger lateral penumbras mean a worse dose distribution.^10^, ^11^ To avoid these issues, a variety of useful methods, such as advanced optimize method (contour scanning),^12^ improvement of beam line,^13^ and various type of aperture or collimator,^14^, ^15^, ^16^, ^17^ have been proposed. In the same way, methods to reduce spot size owing to close the bolus or nozzle to patient were also reported.^18^, ^19^ These methods enable to achieve a sharp lateral penumbra, better dose distribution, and lower out‐of‐field dose. Although various useful methods were reported in recent years, we started scanning treatment with a patient‐specific aperture system (PSAS) in May 2016 to irradiate shallow regions and to obtain sharp lateral penumbras. The PSAS has some downsides that require patient‐specific manufacture and the weight of aperture is a burden to therapists. However, the treatment procedures and QA of the PSAS methods were the same as those with non‐PSAS methods. Furthermore, PSAS methods do not require complicate equipment and machine maintenance such as MLC, so it is easy to apply PSAS in a clinical setting. In the previous study, we presented verification results of the PSAS for the spot scanning beam and the results showed that PSAS reduced the lateral penumbra by 30%–70% in the simple case.^9^ The PSAS can be used with both single‐field uniform dose (SFUD) and IMPT, but the dosimetric advantage of the patient‐specific aperture for IMPT has not been clarified. In this study, we clarified the dosimetric advantage of the aperture for IMPT using DVH, dose distribution, and some dose indexes.

## 2 MATERIALS AND METHODS

### 2.A Scanning delivery system with PSAS

**Figure 2:**
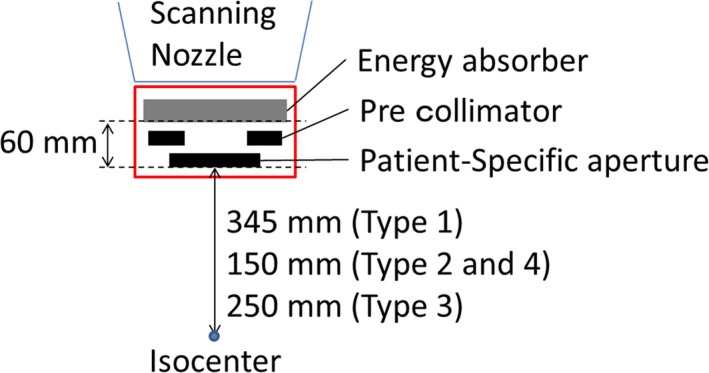
Schematic view of PSAS.

The spot size of the proton beam increases because of scattering by the materials in the beam nozzle and the distance from the exit of beam transport system to the isocenter. Each spot is controlled by scanning magnet and the maximum field size is 30 × 30 cm^2^ in the NPTC. Ninety‐five energies are available for the scanning treatment that are ranging from 71.6 to 221.4 MeV, resulting in water equivalent penetration depths of 4–30.6 g/cm^2^ at intervals of 0.1 g/cm^2^ at low energy beam and 0.6 g/cm^2^ at high energy beam. The irradiation system is able to use a range shifter from 0.1 to 0.5 g/cm^2^ for fine adjustment of the range of high or middle energy beams. The lateral penumbra is generally affected by the distance from the collimator to the surface, depth, beam energy, and energy absorber thickness. Thus, we designed four types of PSAS with different field sizes and distances from the isocenter to put the aperture closer to the patient's body surface. Small PSASs allowed access over the patient's body or patient's immobilization devices for small tumors. The EA had 4 g/cm^2^ water equivalent thickness. The maximum water equivalent penetration with PSAS was restricted to 15 g/cm^2^ because the patient‐specific aperture was made from 3‐cm thick brass. Figure 2 and Table 1 showed schematic view and fundamental parameters of the four types of PSASs. The field sizes of the large PSASs (Types 1 and 2) and small PSASs (Types 3 and 4) were 25 × 25 cm^2^ and 10 × 10 cm^2^, respectively. The distance from the isocenter to the aperture was 150–345 mm. As Types 2 and 4 had the same isocenter‐to‐aperture distance, the spot sizes were the same and the largest spot size was 18.9 mm. Type 1 shows the largest spot size that is 26.7 mm.

**Table 1:** Fundamental parameters of the four types of PSAS

Type	Field size (cm^2^)	Distance from the isocenter (mm)		Maximum spot size (mm)
		To aperture	To absorber	
1	25 × 25	345	405	26.7
2	25 × 25	150	210	18.9
3	10 × 10	250	310	22.9
4	10 × 10	150	210	18.9

### 2.B Treatment planning and patient selection

TPS used in this study was VQA ver. 3.0.1 (Hitachi Ltd.). The VQA is a commercially available TPS in Japan. The VQA was used for spot scanning with or without PSAS. The VQA system uses a pencil beam algorithm with the triple Gaussian (TG) kernel model to improve the accuracy of dose calculation for the scanning beam.^20^ In order to handle the aperture, a fluence dose model^21^ was used. The dose calculation with PSAS has sufficiently high‐accuracy and details of commissioning results were showed in our previous study.^9^

Ten head and neck (HN) tumor cases treated by IMPT technique with the PSAS in NPTC were examined. HN tumors are a suitable region for this scanning method because most HN tumors have a complex geometry and CTV close to organ at risks (OARs). In this study, the target volumes ranged from 14.33 to 260.02 cm^3^ and tumor types were nasal cavity, sinus cavity, mandible, base of skull, and oral cavity. Beam angles of each plan were selected considering clinical conditions such as tumor location, tumor size, and OARs. We created IMPT plans with PSASs for actual patient treatment and without apertures for plan comparison. These plans were optimized separately with the same D_95_ prescription dose and same dose constraint for OAR. The same number of fields and gantry angles were used for each plan and higher priority set to the D_95_ prescription dose. As mentioned earlier, some fields could not use the PSAS because the PSAS has mechanical limitations such as maximum penetration depth, field size, distance from the isocenter and collision with other devices or the patient. In this study, 12 fields could not use PSAS. These 12 fields consisted of four exceeded penetration depth limit fields and eight fields that have mechanical collision between outer frame of PSAS and patient or treatment couch. The treatment plan information is listed in Table 2. All PSAS fields including without aperture fields listed in Table 2 were using the EA. We used a worst case optimization^22^ for dose optimization to obtain the robust plan. Parameters of the worst case optimization were 3 mm for x, y, z direction and 3.5% for range uncertainty,^23^ so 9 scenarios were considered to the target and OARs. The value of 3 mm is the uncertainty of patient setup for HN tumor and machine variability in NPTC. The value of 3.5% is range uncertainty resulting from uncertainties of the range calculation, the acquisition of CT number, and CT number‐Stopping power conversion table. CT images were acquired and reconstructed with 1 mm slices. In the aperture field, the aperture margin was required to assure the marginal dose of the target. The aperture margin was computed by expanding of the maximum outline of the target from the beam's eye view and the margin set to the same value as the spot spacing. The spot spacing was affected by spot size, so the aperture margin was being from 7.2 mm to 11.2 mm at isocenter.

**Table 2:** Plan information with fields, gantry angle, prescribed dose, CTV volume, and OAR

ID	Gantry angle (°)	Aperture type	Field aperture/all	Energy range (MeV)	Prescribed dose (GyE)	CTV volume (cc)	OAR
1	0	Type 2	1/3	142.7–76.2	60.8	258.9	Lens
	120	Non		161.1–71.6			Optic nerve
	240	Non		163.4–71.6			Parotid
2	30	Type 3	3/3	144.4–81.4	70.2	14.3	Parotid
	90	Type 3		135.8–76.2			
	140	Type 4		146.3–77.9			
3	30	Type 4	3/4	135.8–76.2	70.2	145.6	Parotid
	65	Type 3		130.5–76.2			Tongue
	105	Type 1		130.5–77.0			
	195	Non		173.3–107.3			
4	55	Type 3	2/4	137.5–71.6	70.2	58.2	Brain
	305	Type 3		146.3–76.2			Brain stem
	115	Non		163.4–105.9			Chiasm
	260	Non		168.3–104.5			
5	0	Type 3	1/3	148.3–76.2	70.2	260.0	Chiasm
	130	Non		154.8–71.6			Lens
	230	Non		175.7–80.5			Optic nerve
6	0	Type 1	4/4	159–76.2	70.2	150.5	Brain
	300	Type 1		168.3–76.2			Chiasm
	100	Type 4		146.3–76.2			Eye
	260	Type 4		159–76.2			Optic nerve
7	0	Type 4	2/3	146.3–79.7	70.2	45.5	Brain stem
	130	Non		170.8–112.7			Chiasm
	260	Type 1		148.3–96.1			Eye, optic nerve
8	0	Type 4	2/3	154.8–77.9	70.2	60.7	Brain stem
	110	Type 3		165.7–93.5			Chiasm
	240	Non		163.4–81.4			Eye, optic nerve
9	0	Type 2	3/4	144.4–76.2	70.2	137.8	Brain
	40	Type 2		168.3–76.2			Parotid
	180	Non		175.7–107.3			
	320	Type2		168.3–76.2			
10	0	Type 2	1/3	152.6–76.2	70.2	211.5	Brain stem
	120	Non		168.3–71.6			Chiasm
	240	Non		168.3–71.6			Eye, optic nerve

### 2.C Plan evaluation

We compared with‐ and without aperture plans using dose volume histogram (DVH), dose distribution, and some dose indexes. As mentioned earlier, we made with‐ or without aperture plans with the same target dose so that the 95% dose to the target was equal. In this study, we used relative reduction of some dose indexes such as volume receiving 50% of the prescribed dose (V_50_) and maximum dose (D_max_) or mean dose (D_mean_) to the OARs. V_50_ was used for estimate to the out‐of‐field dose. The relevant OARs of each plan were varied so we evaluated the relevant OARs as described in Table 2. Lens, optic nerve, brain stem, chiasm, and eye were analyzed using D_max_. Parotid, tongue and brain were analyzed by D_mean_.

## 3 RESULTS

**Figure 3:**
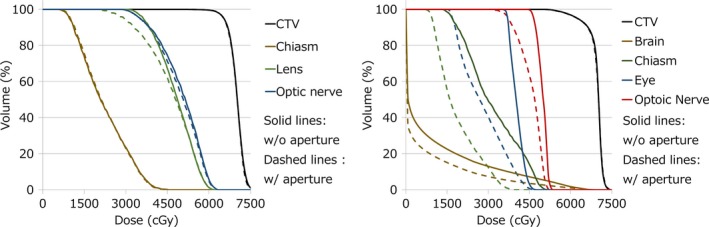
Examples of DVHs of an effective case (left) and an ineffective case (right). Left example is plan ID 6 and right example is plan ID 5. Solid lines are without (w/o) aperture and dashed lines are with (w/) aperture.

**Figure 4:**
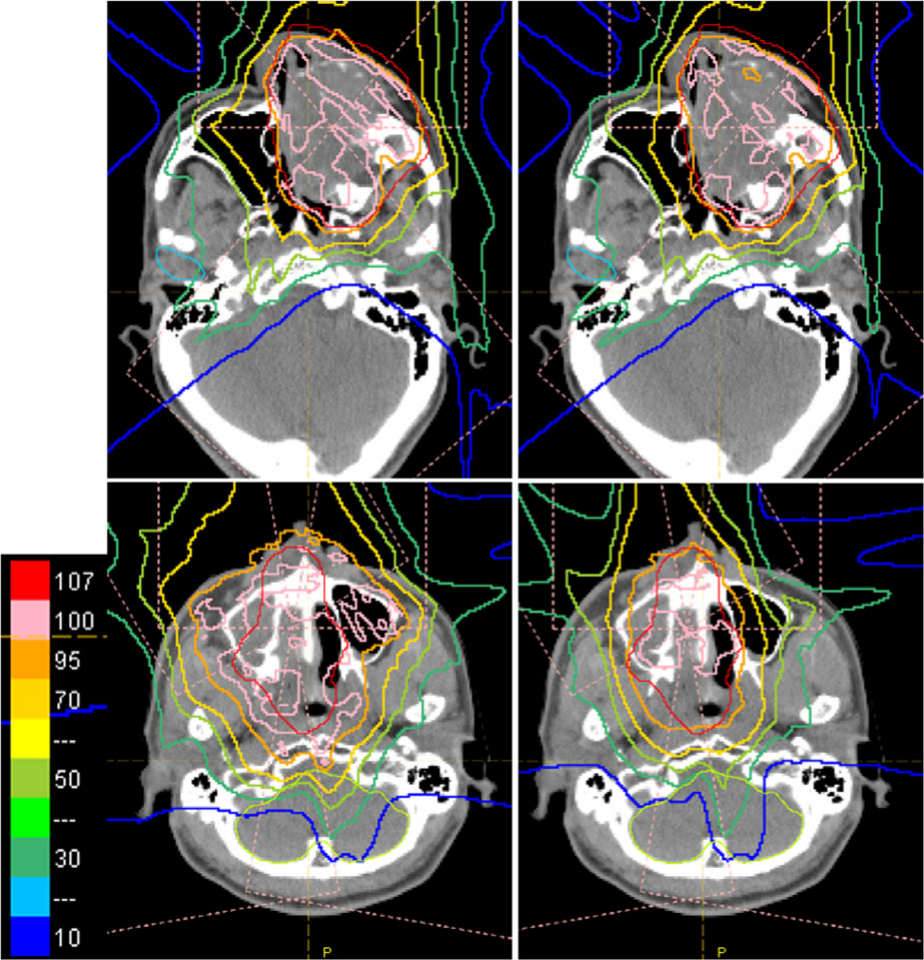
Examples of dose distribution of the ineffective case (upper) and effective case (lower). Upper example is plan ID 5 and lower example is plan ID 6. Right side of each case is with aperture and left side is without aperture. There are same plans shown in Fig. 3.

**Figure 5:**
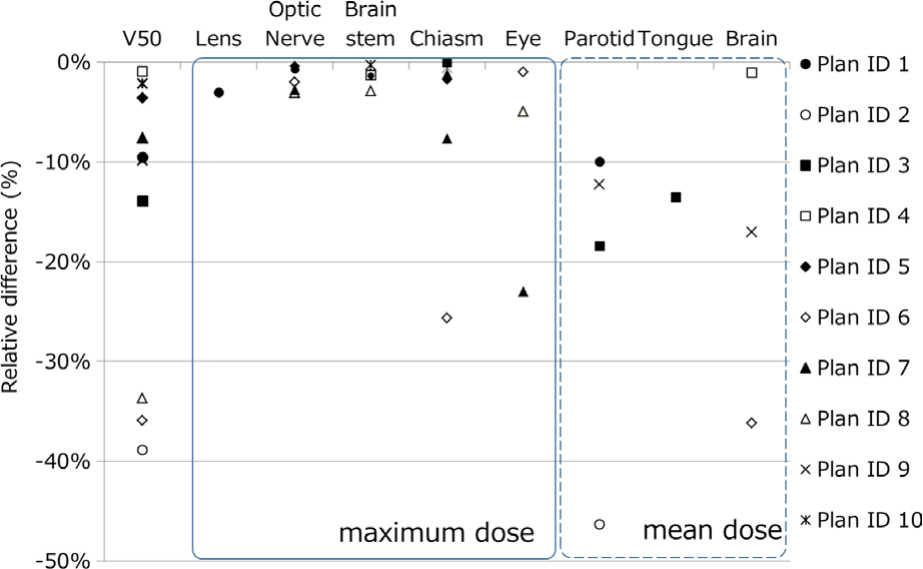
Relative reductions of V_50_, D_max_, and D_mean_ OAR doses between with and without aperture.

Figure 3 shows examples of DVHs with and without aperture. The results show that the target doses were almost equal and some OARs doses were reduced. The most effective case was plan ID 6, in which V_50_ was decreased by 35.9%. In contrast, in plan ID 5, an ineffective case, V_50_ was decreased by only 3.6%. Figure 4 shows examples of dose distribution for both effective and ineffective cases. In the effective case of Fig. 4, all fields can use aperture and apertures are close to the patient surface, as results of which these plans have obviously sharp penumbras. Figure 5 shows the relative difference of V_50_ and D_max_ or D_mean_ between with and without aperture. All examples verified in this study showed decreased V_50_ and OAR doses. The average, maximum, and minimum relative reductions of V_50_ were 15.4%, 38.9%, and 1.0%, respectively. The relative reductions of V_50_, D_max_, and D_mean_ are summarized in Fig. 5. D_max_ and D_mean_ of OARs were decreased by 0.3% to 25.7% and by 1.0% to 46.3%, respectively. Plan IDs 2, 3, 6–9 used apertures for more than half of fields and decreased V_50_ or OAR doses more than 10%. In plan ID 6, D_max_ of the chiasm decreased from 51.3 to 38.2 GyE (25.7%) and D_mean_ of the brain was decreased by 36.2%. The relative reduction of parotid D_mean_ in plan ID 3 was 45.3%. The dose constraints of the OARs of these plans were only fulfilled using aperture plans. At the same time, our PSASs have a maximum penetration depth and mechanical interference limitations so we could not use apertures for all fields. Plan IDs 1, 4, 5, and 10 could only use apertures for not more than half of fields because of the tumor depth or mechanical collision, so these cases showed only small effects.

## 4 DISCUSSION

This study investigated the dosimetric advantages of the patient‐specific aperture to HN IMPT. The patient‐specific aperture reduced V_50_ and OAR doses as shown in Figs. 3, 4, 5 even when using IMPT. Plan dose constraints of ID 2, 6, 7 only fulfilled with the aperture plan and other plans can reduce unnecessary out‐of‐field dose. The decrease rate of D_max_ in some cases was dramatic but other cases showed small effects because OARs were close to the CTV. In this study, we optimized plans while prioritizing the target dose so many maximum dose OARs showed minimal change. In addition, the aperture only collimates the outer line of the target from each beam's eye view and the current optimization algorithm of our TPS cannot consider OAR blocking when using the aperture. In the IMPT plan, the results might be improved by using OAR blocking with an aperture for each field. From the results of our study, using more than half of fields was needed to obtain effective results.

We clarified the dosimetric advantage of patient‐specific apertures with IMPT in many cases. However, PSAS requires patient‐specific manufacture, mounting during treatment and storage of the aperture after treatment, so it was not the best way from the view point of patient throughput and a burden on therapists, too. In addition, some fields could not use PSAS due to mechanical collision and limitation of penetration depth. To overcome these limitations, it is assumed that a movable nozzle and MLC system would improve the lateral penumbra and treatment throughput. Some additional methods have been investigated, for example, to use dynamic collimation with MLC (layer‐by‐layer collimation)^24^ or dynamic collimation (spot‐by‐spot collimation).^16^, ^25^ These methods will come into practical and might improve the dose distribution more. But at present, PSAS system showed dosimetric advantage for shallow region treatment, and in some case, it enables to fulfill dose constraints.

## 5 CONCLUSION

We herein reported improvement of dose distribution by using patient‐specific apertures with IMPT for shallow depth tumor. The PSAS has some dosimetric advantages for clinical use and is easy to use because it does not require complex machine or control mechanism. Using the PSAS has some demerits from the viewpoint of patient throughput and usability; however, it is useful for clinical application.

## CONFLICT OF INTEREST

The authors have no conflicts of interest directly relevant to the content of this article.
